# Spatial heterogeneity of socio-economic determinants of typhoid/paratyphoid fever in one province in central China from 2015 to 2019

**DOI:** 10.1186/s12889-023-15738-0

**Published:** 2023-05-22

**Authors:** Xiang Ren, Siyu Zhang, Piaoyi Luo, Jin Zhao, Wentao Kuang, Han Ni, Nan Zhou, Haoyun Dai, Xiuqin Hong, Xuewen Yang, Wenting Zha, Yuan Lv

**Affiliations:** 1 Key Laboratory of Molecular Epidemiology of Hunan Province, School of Medicine, Hunan Normal University, Changsha, 410013 Hunan China; 2 Hunan Provincial Center for Disease Control and Prevention, Changsha, 410005 Hunan China; 3 Changsha Center for Disease Control and Prevention, Changsha, 410024 Hunan China; 4 Hunan Provincial People’s Hospital, The First Affiliated Hospital of Hunan Normal University, Changsha, 410007 Hunan China

**Keywords:** Typhoid and paratyphoid, Socio-economic determinants, Geographic detector, MGWR model, Spatial heterogeneity

## Abstract

**Background:**

Typhoid fever and paratyphoid fever are one of the most criticial public health issues worldwide, especially in developing countries. The incidence of this disease may be closely related to socio-economic factors, but there is a lack of research on the spatial level of relevant determinants of typhoid fever and paratyphoid fever.

**Methods:**

In this study, we took Hunan Province in central China as an example and collected the data on typhoid and paratyphoid incidence and socio-economic factors in 2015–2019. Firstly spatial mapping was made on the disease prevalence, and again using geographical probe model to explore the critical influencing factors of typhoid and paratyphoid, finally employing MGWR model to analysis the spatial heterogeneity of these factors.

**Results:**

The results showed that the incidence of typhoid and paratyphoid fever was seasonal and periodic and frequently occurred in summer. In the case of total typhoid and paratyphoid fever, Yongzhou was the most popular, followed by Xiangxi Tujia and Miao Autonomous Prefecture, Huaihua and Chenzhou generally focused on the south and west. And Yueyang, Changde and Loudi had a slight increase trend year by year from 2015 to 2019. Moreover, the significant effects on the incidence of typhoid and paratyphoid fever from strong to weak were as follows: gender ratio(q = 0.4589), students in ordinary institutions of higher learning(q = 0.2040), per capita disposable income of all residents(q = 0.1777), number of foreign tourists received(q = 0.1697), per capita GDP(q = 0.1589), and the *P* values for these factors were less than 0.001. According to the MGWR model, gender ratio, per capita disposable income of all residents and Number of foreign tourists received had a positive effect on the incidence of typhoid and paratyphoid fever. In contrast, students in ordinary institutions of higher learning had a negative impact, and per capita GDP shows a bipolar change.

**Conclusions:**

The incidence of typhoid and paratyphoid fever in Hunan Province from 2015 to 2019 was a marked seasonality, concentrated in the south and west of Hunan Province. Attention should be paid to the prevention and control of critical periods and concentrated areas. Different socio-economic factors may show other directions and degrees of action in other prefecture-level cities. To summarize, health education, entry-exit epidemic prevention and control can be strengthened. This study may be beneficial to carry out targeted, hierarchical and focused prevention and control of typhoid fever and paratyphoid fever, and provide scientific reference for related theoretical research.

## Background

Typhoid fever is an infectious disease caused by Salmonella enteric subtype typhoid. Paratyphoid Fever is a disease similar to typhoid fever caused by Salmonella enteric subtype typhoid serotype A, B or C. Typhoid and paratyphoid fever is commonly known as enteric fever (EF), which usually presents with febrile symptoms accompanied by chills. Most patients also present with gastrointestinal reactions such as abdominal pain, constipation and diarrhea, and a few patients present with rose spots, which are usually difficult to distinguish from other acute febrile diseases [1]. The mortality of enteric fever is low and the course of the disease is short, however, if the clinical symptoms are not treated in time, complications are likely to occur, such as encephalopathy, gastrointestinal bleeding and perforation, which may lead to disability and even threaten life [2]. The disease is transmitted through the fecal–oral route and is easily transmitted through water or food contaminated with feces [3].

Typhoid and paratyphoid fever are still one of essential public health problems in the world. Studies estimated that there were 14.3 million cases of typhoid and paratyphoid fever, with 135,900 deaths, resulting in 9.8 million disability-adjusted life years (DYs) in 2017 [4]. It is worth noting that in the context of economic globalization, the flow of travelers from different places has contributed to the spread of the disease. Typhoid and paratyphoid are second only to malaria as potentially life-threatening travel-related infectious diseases [5, 6], and the largest-ever outbreak of paratyphoid. A occurred on an airplane of Israeli travelers in Nepal. Vaccines are an effective means to prevent infectious diseases, but nowadays, new generation of typhoid conjugate vaccines (TCVS) are in various stages of development and licensing [7]. Even worse, there is no vaccine to prevent paratyphoid fever, and several paratyphoid vaccine candidates are in the early stages of development.

Globally, typhoid and paratyphoid fever are most prevalent in developing countries, especially low- and middle-income countries (LMICs), where the corresponding disease burden is also high. Studies have shown that during 1990–2018, the most significant typhoid and paratyphoid outbreaks occurred in Asia, followed by Africa, Oceania and a few outbreaks in Europe and North America [8]. China as a part of Asia, where typhoid and paratyphoid are widespread. For many years, the incidence of typhoid and paratyphoid fever in Yunnan Province has ranked first in China. And studies in the province have found that Xishuangbanna Prefecture [9], Yuxi Prefecture and the border area between Myanmar and Laos [10] are high incidence areas. Besides, Zhejiang Province is also one of the places with a increased incidence of typhoid and paratyphoid fever where a total of 16 outbreaks of typhoid and paratyphoid fever were reported from 1953 to 2013 (5 typhoid and 11 paratyphoid A). As the study area, Hunan Province reported 9 888 typhoid and paratyphoid cases from 2011 to 2020, with an average annual incidence of 1.46/100 000 [11]. Both 2009–2013 [12] and 2015–2016 [13] were at the forefront of typhoid and paratyphoid fever in China, so it is necessary to prevent and control typhoid and paratyphoid fever in this region.

At present, domestic and foreign related shows that the incidence of this disease may be closely related to socio-economic factors. A study in Taiwan showed that age and gender were important factors affecting the incidence of typhoid and paratyphoid fever, with the most cases occurring between the ages of 20 and 40 years from 2011 to 2020, and the infection rate of females was higher than that of males [14]. Other socio-economic factors also affect the incidence of typhoid and paratyphoid fever, such as population density, literacy rate, unemployed population, etc. [15] or personal hygiene habits and household hygiene conditions, etc. [16]. The subject on which this paper is based, spatial epidemiology, is an important branch of epidemiology. As early as the London cholera outbreak in 1854, Professor John Snow plotted cases on a map of London, and the outbreak was quickly controlled by closing Wells in areas where cases were concentrated. In the past decades, the application of spatial epidemiology in infectious diseases has been developing continuously and has a substantial development prospect [17]. All kinds of infectious diseases can be well integrated with spatial epidemiology to achieve the purpose of strengthening the prevention and control of the disease, For example, hand, foot and mouth disease [18], schistosomiasis [19], malaria [20], dengue fever [21], etc. The application of COVID-19 in spatial epidemiology is now one of the hot spots [22–24]. In recent years, the study on the spatial epidemiology of typhoid and paratyphoid fever is rare, the same goes for the study on the social influencing factors using spatial models.

Therefore, this paper collected the incidence data of typhoid and paratyphoid fever in Hunan Province from 2015 to 2019 and the socio-economic data for the same period. Descriptive epidemiology was used to describe the time distribution of the disease, spatial epidemiology was used to find out the hot spots of the disease, and spatial geographical models were used to explore the vital epidemic factors of the disease. This study may achieve the targeted prevention of typhoid and paratyphoid, and provide scientific reference for theoretical research.

## Methods

### Study area

**Fig. 1:**
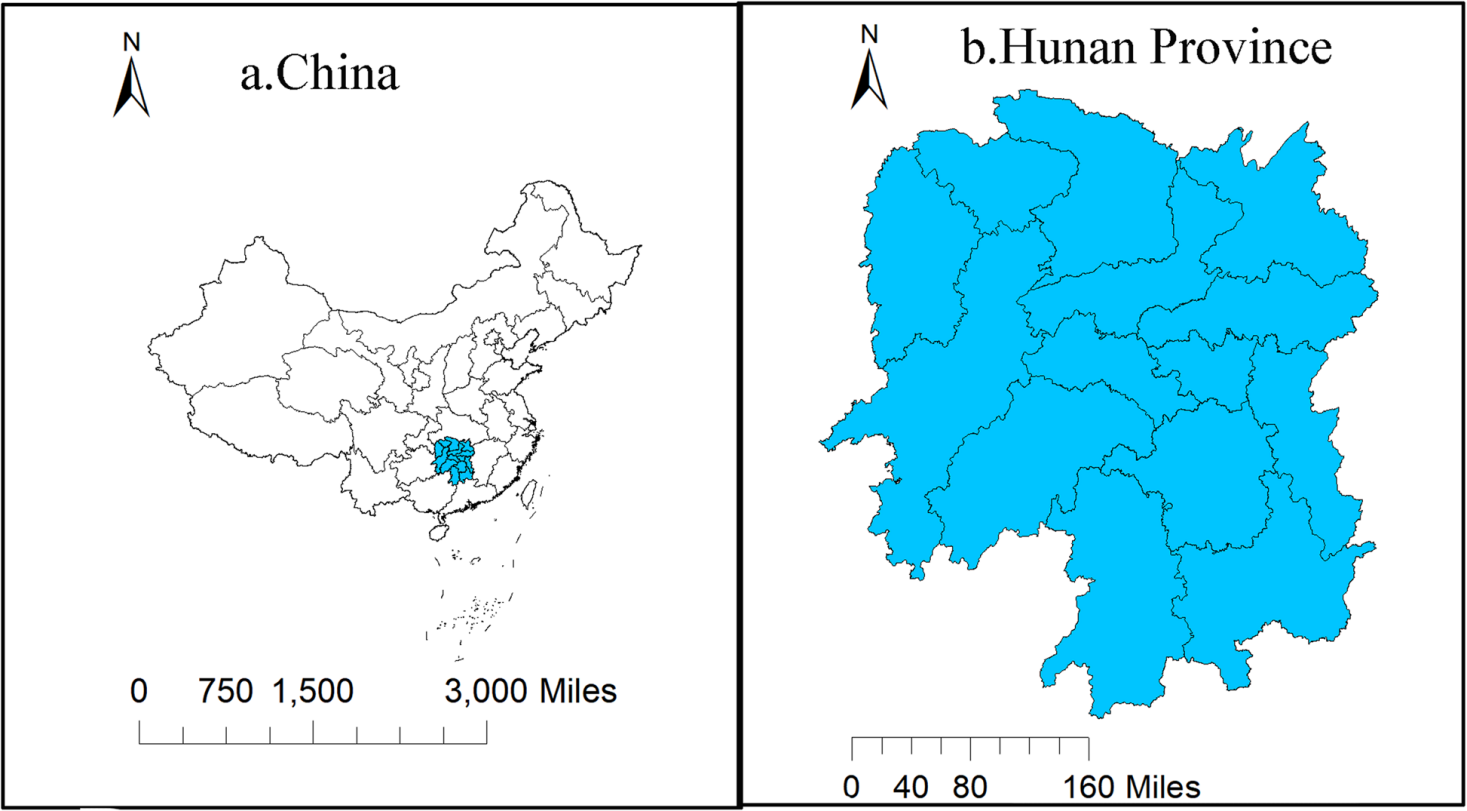
Schematic diagram of study area

The area studied in this paper is Hunan Province (Fig. 1), which is located in the middle of China, 108°47 '-114°15' E, 24°38 '-30°08' N. It is named "Hunan" because most of the area is in the south of Dongting Lake. Hunan is a subtropical monsoon climate, cold winter and hot summer, variable spring and autumn temperatures. There are dense river networks, developed river systems and various landforms mainly mountains and hills. As of December 31, 2021, the province has jurisdiction over 13 prefecture-level cities and 1 autonomous prefecture, which are Changsha, Zhuzhou, Xiangtan, Hengyang, Shaoyang, Yueyang, Changde, Zhangjiajie, Yiyang, Chenzhou, Yongzhou, Huaihua, Loudi and Xiangxi Tujia and Miao Autonomous Prefecture.

### Data sources

The incidence data of typhoid fever and paratyphoid fever in 13 prefecture-level cities and 1 autonomous prefecture in Hunan Province from 2015 to 2019 were obtained from Hunan Center for Disease Control and Prevention. The economic and social factors in the influencing factors come from the statistical yearbook of Hunan Province. Based on data availability, biological characteristics of typhoid/paratyphoid fever and previous studies, economic and social factors include demographic characteristics (gender ratio, etc.), economic level (per capita GDP, per capita disposable income of all residents, etc.), education and culture (students in ordinary institutions of higher learning, education spending, etc.), urban development(urbanization rate, employment, etc.), health resources number (health care institutions, health technicians, etc.), tourism (total number of tourists received, number of foreign tourists received, etc.).

### Research framework

On the one hand, the temporal and spatial characteristics of typhoid and paratyphoid fever in Hunan Province were analyzed. The key prevention and control periods of typhoid and paratyphoid fever were obtained by making the temporal trend chart of the incidence. With the help of GIS system, the spatial map of the disease was made to find the different spatial distribution characteristics of the disease, and the hot spots were obtained in order to strengthen the key prevention and control in the region.

**Fig. 2:**
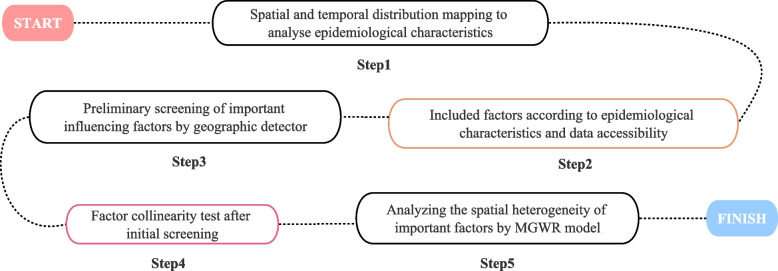
Schematic diagram of the technical route of the study

On the other hand, the geospatial model was used to explore and analyze the influence of social environment on typhoid and paratyphoid fever. Firstly, the influential socio-economic factors were preliminarily explored by using factor detection in geographic detector. Next, on the basis of previous step, the included factors will be tested for collinearity by VIF test (variance inflation factor test). Generally speaking, it indicates a strong collinearity relationship if VIF exceeds 10. Then the final influencing factors were included and brought into the MGWR model to analyze the spatial heterogeneity of important influencing factors (different influencing factors play different roles in different regions) (Fig. 2). All methods were carried out in accordance with relevant guidelines.

### Statistical analysis

#### Geographical detector

**Fig. 3:**
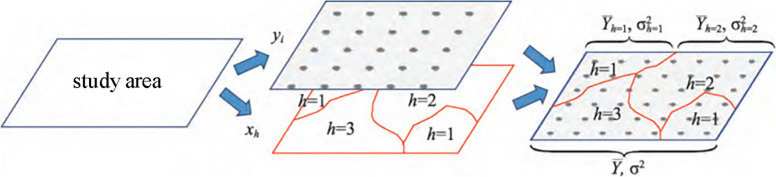
Schematic diagram of factor detector principle

In this study, factor detector in geographic detector is used to explore the spatial variability of attribute y explained by a certain factor x (Fig. 3), which is mainly measured by q statistic, and its formula is as follows:

1 $$q=1-\frac{\sum\limits_{h=1}^{L}{N}_{h}{{\sigma }^{2}}_{h}}{N{\sigma }^{2}}=1-\frac{SSW}{SST}$$

Where: h is the number of classification partitions; N_h_ and N are the number of units in the class and the whole region; SSW is the sum of the class variances, and SST is the total variance of the entire region. The value range of q is [0,1], and the larger the value, the greater the explanatory power of factor x to attribute y. Factor detector has many advantages such as: based on nonlinear assumption; collinearity can be avoided; both qualitative and quantitative factors can be analyzed.

#### MGWR model

The MGWR model was first proposed by Fotheringham in 2017, and the statistical inference method of this model was obtained by Yu et al. in 2019. The classical GWR model (geographic weighted regression model) can bring the quantitative relationship between the dependent variable and the independent variable that varies with space. Notably, all the independent variables in the model can only accept a unified scale of action in MGWR model, which can more accurately and genuinely reflect the quantitative relationship between independent variables and dependent variables at the spatial level. The structure of MGWR model is as follows:

2 $${y}_{i}={\beta }_{0}\left({u}_{i},{v}_{i}\right)+\sum_{j=1}^{k}{\beta }_{bwj}\left({u}_{i},{v}_{i}\right){x}_{ij}+{\varepsilon }_{i}$$

Where: y_i_ is the attribute value at i; (u_i_,v_i_) is the coordinate at position i; β_0_(u_i_,v_i_) is the intercept of the model at i; bwj represents the bandwidth used by the j^th^ variable regression coefficient; β_bwj_(u_i_,v_i_) is the regression coefficient of the j^th^ variable at i; Ɛi is the error term of the model at i. The model kernel function and the broadband selection criterion adopted in this paper are the quadratic kernel function and the corrected Akaike information criterion (AICc) respectively, and the determination coefficient and Akaike information criterion are the indexes used in the comparison of the three models.

## Results

### Time and space distribution

**Fig. 4:**
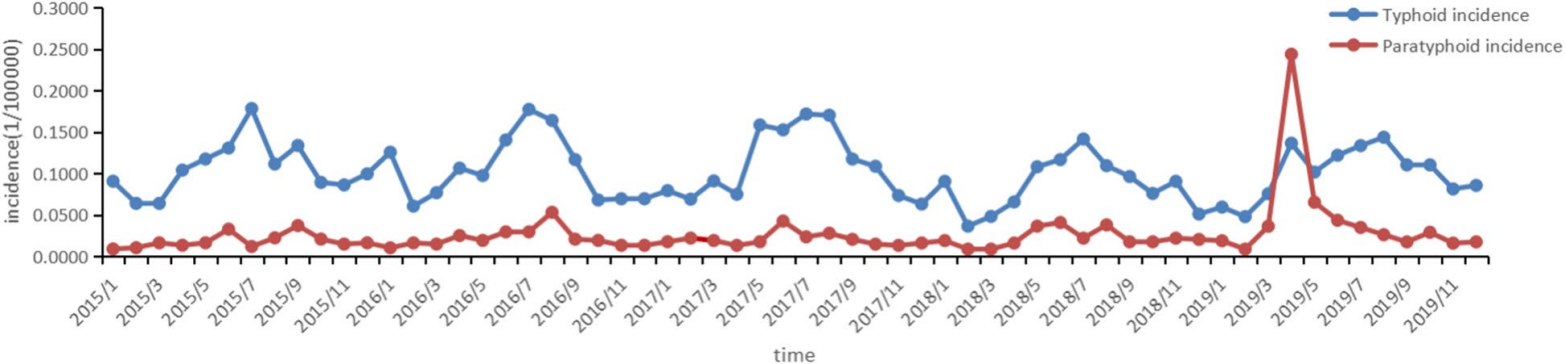
Temporal distribution of typhoid and paratyphoid in Hunan Province, 2015–2019

The time trend of the monthly incidence of typhoid and paratyphoid fever in Hunan Province from 2015 to 2019 was shown in the following Fig. 4: The incidence of typhoid and paratyphoid fever was cyclical and obviously seasonal, concentrated in summer, rising roughly from March and April, reaching the peak in July and August, and then showing a gradual downward trend every year. From 2015 to 2019, the incidence of typhoid fever was higher than that of paratyphoid fever, except in April 2019, when the incidence of paratyphoid fever exceeded the incidence of typhoid fever at the highest level. The annual trend of the incidence of typhoid and paratyphoid fever in Hunan Province from 2015 to 2019 was similar, and the annual peak of typhoid fever incidence showed a slightly downward trend. The incidence of paratyphoid fever in 2019 was higher than that in other years.

**Fig. 5:**
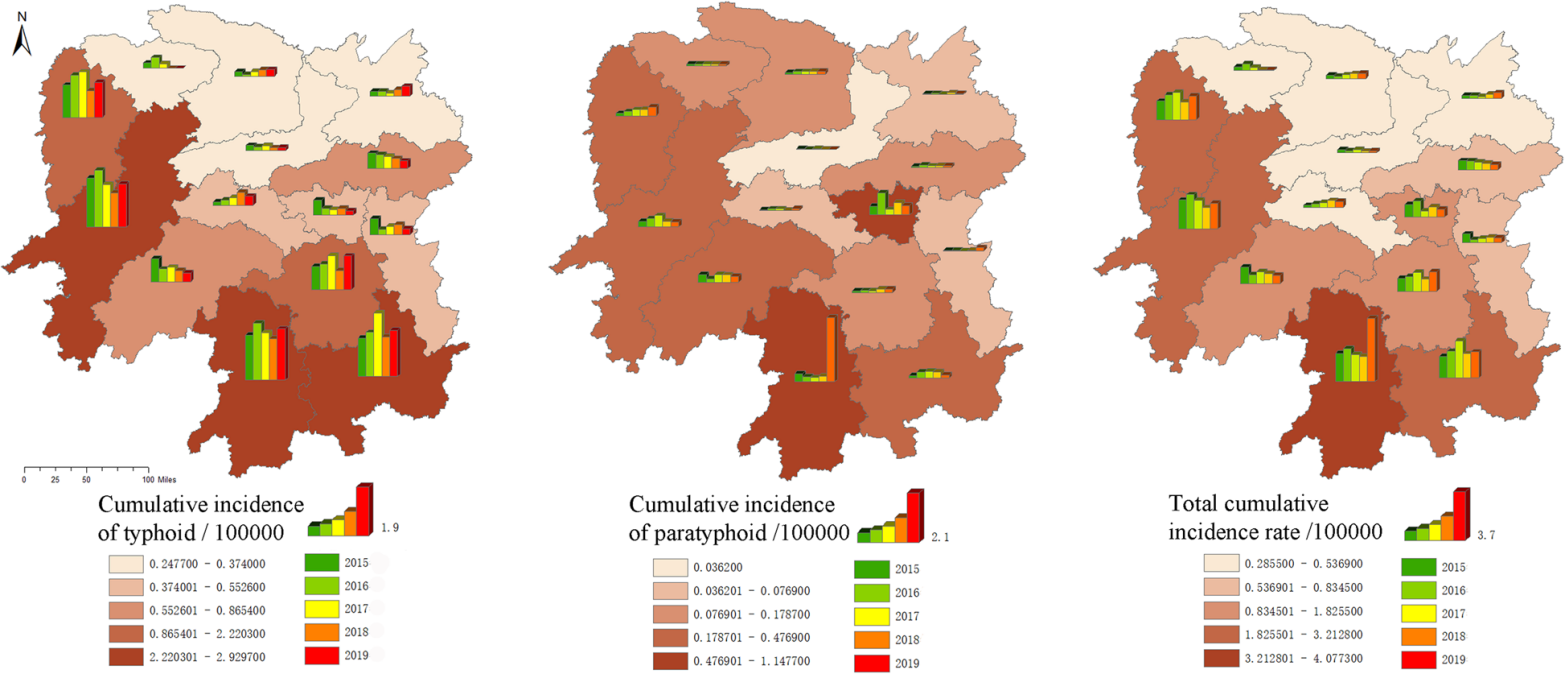
Spatial distribution of typhoid and paratyphoid in Hunan Province, 2015–2019

The spatial distribution characteristics of typhoid and paratyphoid in Hunan Province from 2015 to 2019 were shown in the Fig. 5. The typhoid cases were mainly concentrated in Huaihua, Yongzhou and Chenzhou, followed by Hengyang and Xiangxi Tujia and Miao Autonomous Prefecture. From 2015 to 2019, the incidence of typhoid fever in most prefecture-level cities showed irregular and small fluctuations, with a slight upward trend in Yueyang and Loudi. Paratyphoid fever mainly occurred in Yongzhou and Xiangtan, followed by Xiangxi Tujia and Miao Autonomous Prefecture, Huaihua, Shaoyang and Chenzhou. The incidence of paratyphoid fever fluctuated little from 2015 to 2019, but the incidence of paratyphoid fever increased sharply in Yongzhou in 2019. In the case of typhoid and paratyphoid fever, the most common cases occurred in Yongzhou, followed by Xiangxi Tujia and Miao Autonomous Prefecture, Huaihua and Chenzhou. In general, the cases were concentrated in the south and west of Hunan Province, with the lowest incidence in the north. From 2015 to 2019, the incidence of most prefecture-level cities except Yongzhou had a small fluctuation range, and Yueyang, Changde and Loudi had a slight increase trend year by year.

### Screening of important influencing factors

**Fig. 6:**
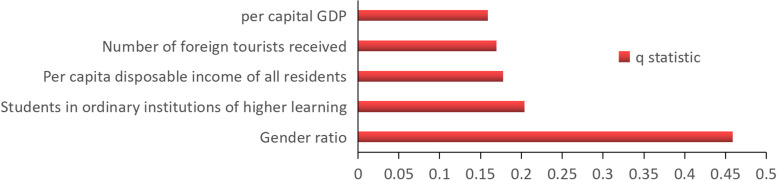
Factor screening results of factor detector

Based on the data availability, typhoid paratyphoid biology and past research, we obtained the various aspects of social environment factors into form, first using the K-means to partition, multiple factors which the data and consolidation after the input in Excel geographic detector, several important influencing factors, it is concluded that there were statistically significant Specific results were shown in the figure below: important factor of typhoid paratyphoid incidence influence from strong to weak: gender ratio (male per 100 women), students in ordinary institutions of higher learning (person), per capita disposable income of all residents (yuan), number of foreign tourists received (person), per capita GDP (yuan), and these corresponding *P* values are less than 0.001(Fig. 6, Table 1).

**Table 1:** q values and corresponding *P* values in factor detector results

	Per capital GDP	Number of foreign tourists received	Per capita disposable income of all residents	Students in ordinary institutions of higher learning	Gender ratio
q statistic	0.1589	0.1697	0.1777	0.2040	0.4589
*P* value	0.000	0.000	0.000	0.000	0.000

### Spatial heterogeneity of important influencing factors

The screened factors were tested for collinearity, as shown in the Table 2, VIF values were all less than 10, tolerance (the reciprocal of VIF) was all less than 1, and they passed the collinearity test.

**Table 2:** Collinearity test results before inclusion

Variables	Per capital GDP(X1)	Number of foreign tourists received(X2)	Per capita disposable income of all residents(X3)	Students in ordinary institutions of higher learning(X4)	Gender ratio(X5)
VIF	2.162	1.996	3.734	5.333	2.477
Tolerance	0.462	0.501	0.268	0.188	0.404

**Fig. 7:**
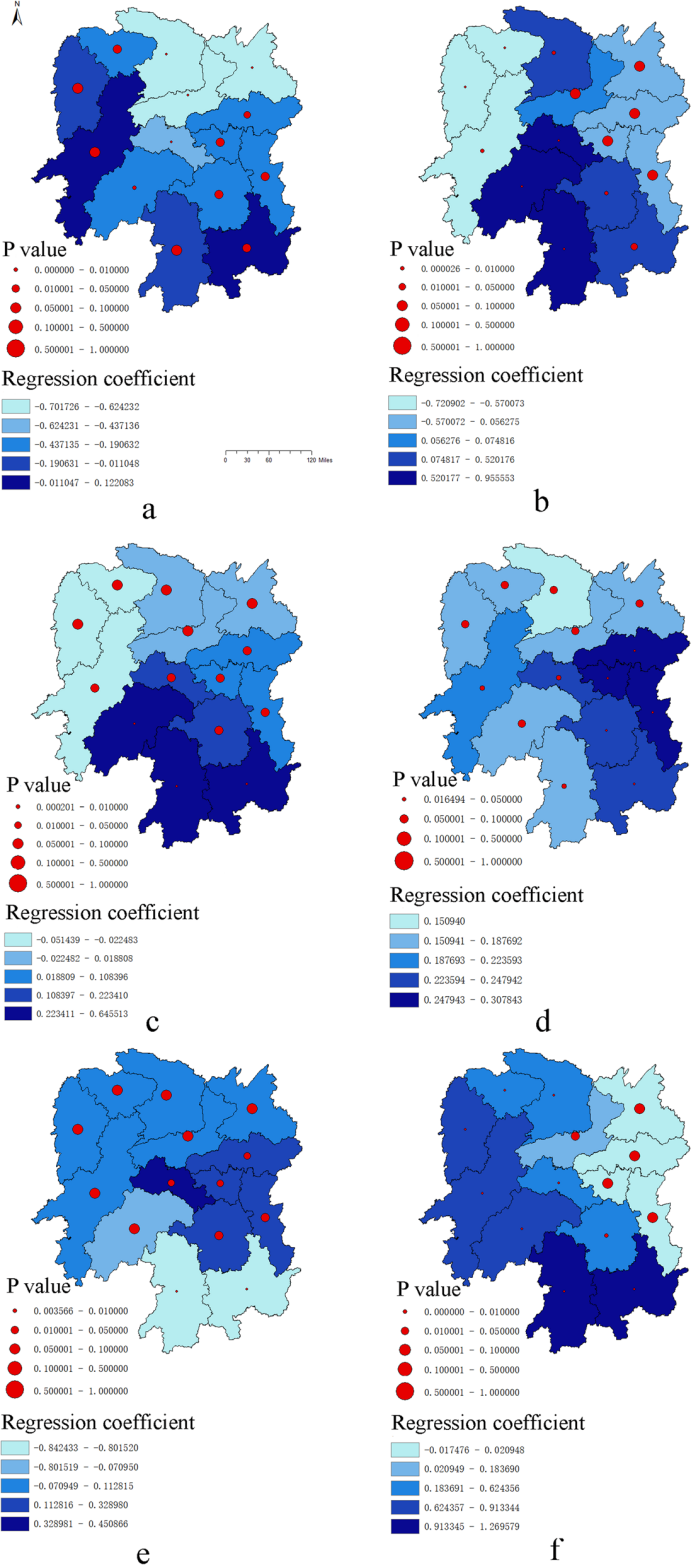
The spatial heterogeneity of typhoid and paratyphoid disease caused by social factors. Note: The factors represented by a and f are Intercept, Per capital GDP (X1), Number of foreign tourists received (X2), Per capita disposable income of all residents (X3), Students in ordinary institutions of higher learning (X4), Gender ratio (X5)

The regression coefficients and the spatial distribution of statistical significance of the influencing factors of typhoid and paratyphoid fever were shown in the Fig. 7. The statistical description of the regression coefficients of each influencing factors were shown in the Table 3.

**Table 3:** Summary statistics for MGWR parameter estimates

Variable	Mean	STD	Min	Median	Max
Intercept	-0.256	0.253	-0.702	-0.208	0.122
Per capital GDP(X1)	0.169	0.529	-0.721	0.066	0.956
Number of foreign tourists received(X2)	0.160	0.210	-0.051	0.105	0.646
Per capita disposable income of all residents(X3)	0.221	0.050	0.151	0.206	0.308
Students in ordinary institutions of higher learning(X4)	0.027	0.376	-0.842	0.057	0.451
Gender ratio(X5)	0.527	0.428	-0.017	0.571	1.270

The constant term, as a whole, was significant in the western region of Hunan Province, and it was a low-value region, and the incidence of typhoid and paratyphoid fever was basically similar to Hunan Province in space, with the lowest incidence in the western region. Per capita GDP(X1), within the scope of Hunan Province, presents a bipolar change. Among them, it had a significant negative effect in Xiangxi Tujia and Miao Autonomous Prefecture, Zhangjiajie and Huaihua, and a strong positive effect in Changde, Hengyang, Loudi, Shaoyang and Yongzhou. Number of foreign tourists received (X2) had a positive impact on the incidence of typhoid and paratyphoid fever, and Shaoyang, Yongzhou and Chenzhou had statistical significance. Per capita disposable income of all residents (X3) showed a significant positive effect in Changsha, Xiangtan, Zhuzhou, Hengyang and Chenzhou. Students in ordinary institutions of higher learning(X4) had a significant negative effect in Yongzhou and Chenzhou. However, the mean value of the regression coefficient of this index in the table was positive, because it did not take into account that there was no statistical difference in the regression coefficient of most regions when the test level was 0.05, and the negative effect of this factor on the incidence was small. Gender ratio (X5) had the largest regression coefficient among all the factors, and had a significant impact on the incidence. The regression coefficient of this factor showed a strong and significant positive effect at the spatial level, with the strongest in Yongzhou and Chenzhou, followed by Xiangxi Tujia and Miao Autonomous Prefecture, Huaihua, Shaoyang, Zhangjiajie, Changde, Loudi and Hengyang. 

## Discussion

Typhoid and paratyphoid fever is still a long way to go before the disease is completely eliminated. Relevant studies have shown that it will take at least several decades [25]. The aim of this paper is to effectively prevent and control typhoid and paratyphoid fever, and explore the epidemiological rules of this disease in Hunan Province by using space technology in spatial information system. The specific result and analysis were as follows:

By analyzing the time of the incidence of typhoid and paratyphoid fever, we found that the onset of typhoid and paratyphoid fever accumulated in summer with apparent periodicity and seasonality. Some studies also found that typhoid and paratyphoid fever frequently occurred in summer [26], and the further away from the equator, the more pronounced the seasonal change was [27]. Meanwhile the incidence of disease in Hunan Province raised between March and April, reached its peak in July and August, and then showed a gradual downward trend. From 2015 to 2019, the overall incidence of typhoid fever was higher than that of paratyphoid fever. The annual peak of typhoid fever incidence showed a slightly decreasing trend, and the incidence of paratyphoid fever in 2019 was higher than that in other years. The large fluctuation of paratyphoid fever may be related to outbreaks during the period, which mainly occurred in schools and were correlated to water pollution [12]. Therefore, the management of drinking water in schools should be strengthened. In conclusion, Hunan Province should support the prevention and control of typhoid fever in the overall trend, and the prevention and control of paratyphoid fever should not be ignored, mainly because there is no targeted vaccine for paratyphoid fever so far.

Through spatial analysis of the incidence of typhoid and paratyphoid fever, we found that the incidence of typhoid fever in Hunan Province from 2015 to 2019 was mainly concentrated in Huaihua, Yongzhou and Chenzhou, and Yueyang and Loudi had a slight upward trend. The incidence of paratyphoid fever was concentrated in Yongzhou and Xiangtan, with little fluctuation in most areas. However, the incidence of paratyphoid fever increased in Yongzhou in 2019. Typhoid and paratyphoid fever mainly occurred in Yongzhou, followed by Xiangxi Tujia and Miao Autonomous Prefecture, Huaihua and Chenzhou. In general, the incidence was concentrated in the south and west of Hunan Province, and Yueyang, Changde and Loudi had a small increase trend year with each passing year. To achieve effective prevention and control, we should pay attention to the prevention and control of the incidence cluster, and prevent the areas with rising incidence trend. The corresponding allocation of health resources can be used as a reference.

After perceiving the temporal and spatial characteristics of typhoid and paratyphoid fever, we also studied the important socio-economic factors affecting the incidence. We put the data of factors into the geographic detector model after sorting out the format, then got the statistically significant influencing factors. Among them, gender ratio has the most decisive impact, followed by students in ordinary institutions of higher learning, per capita disposable income of all residents, number of foreign tourists received and per capita GDP. Given the above, we still cannot comprehend the direction of action and the spatial heterogeneity of these influencing factors, so it is necessary to use the MGWR model for analysis. Through the analysis of MGWR model, we found that:

Per capita GDP showed a bipolar change in Hunan Province. It had a significant negative effect in Xiangxi Tujia and Miao Autonomous Prefecture, Zhangjiajie and Huaihua, while a strong positive effect in Changde, Hengyang, Loudi, Shaoyang and Yongzhou. Per capita disposable income of all residents had a significant positive effect in Changsha, Xiangtan, Zhuzhou, Hengyang and Chenzhou. The above two factors were related to economic level, and the negative effect in some regions may be due to the improvement of living conditions and medical quality caused by the increase of economic level, which led to the decrease in incidence. The positive effect in some areas may be due to the better economic foundation, steady growth in economic status, the economic ability to see a doctor, improvement in disease diagnosis level, and so on, leading to an increase in the disease detection rate, eventually the incidence rate closer to the natural incidence. Research revealed insufficient case detection will occur [28] when febrile patients do not seek medical treatment or do not receive blood testing. The consultation rate and diagnostic level are of great significance for the incidence and prognosis of typhoid and paratyphoid fever.

The number of foreign tourists received had a positive effect that shown in Shaoyang, Yongzhou and Chenzhou. Travel factors are internationally recognized as important factors affecting the incidence of typhoid and paratyphoid fever, especially outbound tourism. The reduced incidence of typhoid and paratyphoid fever during the COVID-19 epidemic in some regions [29] may have something to the reduced number of people entering and leaving the country. Additionally, since most travelers do not receive disease prevention education and vaccination before and after departure [30], the risk of disease exposure is increased. Typhoid and paratyphoid fever are common in Southeast Asia, among which the burden of typhoid and paratyphoid fever in India is weighty and about half of the global deaths are from India [31]. As a part of Asia, China should pay attention to the situation of the disease entering from Southeast Asia and do entry-exit quarantine work.

In Yongzhou and Chenzhou, students in ordinary institutions of higher learning had a significant negative effect. Studies have shown that education level is positively associated with the perception of typhoid infection through food and water [32], which means that the more educated the population, the greater their awareness of how the disease is transmitted, which may ultimately reduce the incidence of the disease.

At the spatial level, gender ratio showed a strong positive effect, with the strongest in Yongzhou and Chenzhou, followed by Xiangxi Tujia and Miao Autonomous Prefecture, Huaihua, Shaoyang, Zhangjiajie, Changde, Loudi and Hengyang, and gender ratio had the greatest effect among all factors. These results suggested that the greater the gender ratio (the greater the number of males), the greater the probability of typhoid and paratyphoid. Cross-sectional studies have found that the incidence rate of males is greater than that of females [33], which may be revelant to male hygiene habits. Poor hygiene is more likely to spread pathogens, so men should develop good hygiene habits, such as washing hands before meals and after using the toilet, and not drinking raw water without treatment.

There were some limitations in this paper. Influenced by data availability, most of the influencing factors in this study were obtained from China Statistical Yearbook. It is better to incorporate more influencing factors from more aspects and angles, so as to get more accurate results. This study was based on the GIS platform, supported by space mapping and spatial geographic model, considering the spatial difference, on the basis of the typhoid paratyphoid in hunan province was obtained space–time characteristics as well as important social influence factors of the disease, to offer a scientific basis for further prevention and control of the disease, at the same time contributing to the theory of scientific research of the disease.

## Conclusions

Incidence of typhoid and paratyphoid fever in Hunan Province from 2015 to 2019 was a marked seasonality, concentrated in the south and west of Hunan Province. The prevention and control of typhoid fever and paratyphoid fever were strengthened in all prefecture-level cities in the region, especially Yongzhou, Xiangxi Tujia and Miao Autonomous Prefecture, Huaihua and Chenzhou. The incidence of typhoid and paratyphoid fever was be closely bound up with many social factors, and the influence of each factor has different spatial heterogeneity. Gender ratio has the most decisive impact, followed by Students in ordinary institutions of higher learning, per capita disposable income of all residents, number of foreign tourists received and per capita GDP. Therefore, the prevention and control capacity could be enhanced in health education, entry-exit epidemic prevention, environmental monitoring and other aspects. Spatial model analysis and infectious diseases can achieve a high degree of integration in spatial epidemiology. This paper provided a scientific basis for the prevention and control of typhoid and paratyphoid fever in Hunan Province. In view of the lack of research in this field, it is hoped that more researches can be done to this end.

## Data Availability

Some of the datasets analyzed in this study are available from the corresponding author on reasonable request.
